# Crosslinking Kinetics of Methylcellulose Aqueous Solution and Its Potential as a Scaffold for Tissue Engineering

**DOI:** 10.3390/polym11111772

**Published:** 2019-10-28

**Authors:** Beata Niemczyk-Soczynska, Arkadiusz Gradys, Dorota Kolbuk, Anna Krzton-Maziopa, Pawel Sajkiewicz

**Affiliations:** 1 Institute of Fundamental Technological Research, Polish Academy of Sciences, Pawinskiego 5b St., 02-106 Warsaw, Poland; argrad@ippt.pan.pl (A.G.); dkolbuk@ippt.pan.pl (D.K.); psajk@ippt.pan.pl (P.S.); 2 Faculty of Chemistry, Warsaw University of Technology, Noakowskiego 3 St., 00-664 Warsaw, Poland; anka@ch.pw.edu.pl

**Keywords:** methylcellulose, thermosensitive hydrogel, crosslinking kinetics, DSC, DMA, cellular tests

## Abstract

Thermosensitive, physically crosslinked injectable hydrogels are in the area of interests of various scientific fields. One of the representatives of this materials group is an aqueous solution of methylcellulose. At ambient conditions, methylcellulose (MC) is a sol while on heating up to 37 °C, MC undergoes physical crosslinking and transforms into a gel. Injectability at room temperature, and crosslinkability during subsequent heating to physiological temperature raises hopes, especially for tissue engineering applications. This research work aimed at studying crosslinking kinetics, thermal, viscoelastic, and biological properties of MC aqueous solution in a broad range of MC concentrations. It was evidenced by Differential Scanning Calorimetry (DSC) that crosslinking of MC is a reversible two-stage process, manifested by the appearance of two endothermic effects, related to the destruction of water cages around methoxy groups, followed by crosslinking via the formation of hydrophobic interactions between methoxy groups in the polymeric chains. The DSC results also allowed the determination of MC crosslinking kinetics. Complementary measurements of MC crosslinking kinetics performed by dynamic mechanical analysis (DMA) provided information on the final storage modulus, which was important from the perspective of tissue engineering applications. Cytotoxicity tests were performed using mouse fibroblasts and showed that MC at low concentration did not cause cytotoxicity. All these efforts allowed to assess MC hydrogel relevance for tissue engineering applications.

## 1. Introduction

Hydrogels are three dimensional polymeric structures, crosslinked by either chemical or physical interactions, forming a polymeric network. They are characterized by a wide range of processing possibilities in tailoring their biological and mechanical properties [1,2]. In this regard, the crosslinking method is essential, when determining the final properties of hydrogel including their stability in physiological conditions. Chemically crosslinked hydrogels consist of permanently formed junctions as a result of chemical reactions. Although the covalent crosslinking results in high stability and increased mechanical properties, the use of toxic crosslinking agents, usually, disqualify their application in tissue engineering. Physical crosslinking is a temporary effect of generation of the junctions, which are related to entanglements of polymeric chain, ionic bonds, hydrogen bonds, or hydrophobic interactions [2,3]. One of the interesting types of materials exhibiting physical interactions are the stimuli-responsive hydrogels. Their crosslinking takes place under an external trigger. In this respect, magnetic (ferrogels), pH-stimuli-responsive or thermosensitive hydrogels are distinguished [4,5]. Especially, the last group has gained much attention in various fields of science [6]. An example of such an approach is methylcellulose (MC)—a polysaccharide, which is a derivative of cellulose. Contrary to pure cellulose, MC is water soluble. This is due to partial substitution of the hydroxyl groups (–OH), which are responsible for the formation of crystal fibers in pure cellulose, with the methoxy groups (–OCH_3_), which prevents crystalline fibers formation [7].

Considering its effective support in cells regeneration [8] and having been approved by the Food and Drug Administration (USA), MC is an attractive material for tissue engineering or drug delivery systems [9]. Contrary to other natural hydrogels (e.g., alginate), MC constitutes a stable structure in physiological conditions, with mechanical properties similar to native tissues (e.g., meniscus, liver, or spinal cord) [10,11,12,13,14]. From the perspective of tissue engineering, the thermosensitive crosslinking of MC aqueous solution seems very promising, as it prevents the use of toxic crosslinking agents [15]. 

The physical crosslinking of MC is a consequence of the formation of hydrophobic domains. In MC aqueous solution, the crosslinking appears during heating near 40 °C [9]. The sol–gel transition is determined by the Lower Critical Solution Temperature (LCST), above which hydrophobic interactions start to prevail in the solution [8]. The LCST is known to depend on the methoxy group substitution degree, MC concentration as well as additives to the MC solution [1]. According to [16], the crosslinking process consists of two stages. At ambient conditions, polymer-solvent interactions between hydrophilic MC groups and water dominate. The “water cages” around the hydrophobic molecules of MC result in weak polymer–polymer interactions. This is a classic example of hydrophobic hydration, preventing crosslinking of MC at room temperature [16]. While the temperature increases near the physiological temperature, water cages are shattered and the hydrophobic regions of MC are exposed leading to stronger polymer–polymer interactions. Higher temperature amplifies the polymeric chains mobility and the methoxy groups get closer to each other and self-organize to form hydrophobic interactions. The partial polymeric chain rearrangement takes place and the primary hydrophobic interactions (non-cooperative) are formed. This step represents the first stage of crosslinking. While the hydrogel is still heating, hydrophobic domains and consequently three-dimensional networks are formed. The remaining bonded MC chains generate supplementary hydrophobic interactions, called cooperative, to increase the network cells’ density. The higher temperature (above 60 °C) stimulates the network cells to uptake and retain water from the solution, which is an effect of osmotic forces. As a consequence, fully swelled, mechanically stable, and a crosslinked network is formed.

Considering the materials for tissue engineering applications, an appropriate kinetics of hydrogel crosslinking is extremely important. On one hand, it should take place slowly enough to allow for a complete injection and fill the injury, but on the other hand, it should be sufficiently fast to prevent it from moving away from the aimed location. The mechanical properties are important to provide an appropriate biomaterial adhesion to the tissue and to avoid pressure and the spreading of the injury among the tissues [17,18]. Crosslinking the kinetics of MC and its final stiffness depends on different parameters of the used product, such as the substitution degree or molecular weight, but it also might be adjusted using various concentrations and additives [19]. In this study, we used one particular type of MC with various concentrations, to determine the effect of the MC concentration on the crosslinking process.

Martin et al. [15] investigated the crosslinking rate of MC using the inverted test tube method. Briefly, the test tube was inverted and if the solution no longer flowed, it was considered to be a solid and the transition time was registered as the crosslinking time. It is worth noticing that this experiment was carried out in a simple manner, providing only the basic macroscopic information about the material. Another commonly used method for MC crosslinking kinetics determination involves rheological measurements (e.g., oscillatory shear measurements) [20,21] and DSC studies [22,23]. Despite the methods showing the dependencies between the MC network changes and the temperature, most of the DSC measurements were just focused on the sol–gel transition temperature or their dependencies, while a certain salt was added to the MC solution.

The objective of our work was to systematically investigate the effect of MC concentration on the crosslinking kinetics of MC water solutions using two complementary methods—dynamic mechanical analysis (DMA) and DSC. DMA provides additional information on the viscoelastic properties of the final hydrogel. Both parameters—crosslinking kinetics and viscoelastic properties are crucial in the determination of the adequate MC concentrations for tissue engineering applications. Additionally, the biological properties of the MC aqueous solution was determined in order to evaluate its significance for tissue engineering application.

## 2. Materials and Methods 

### 2.1. Preparation of MC Aqueous Solution

The MC (METHOCEL A15LV, Sigma Aldrich, St. Louis, MO, USA) solution was prepared in demineralized water at various weight concentrations (0.75–20 wt.%). The procedure was carried out using the “hot–cold” dispersion method, according to [24]. Briefly, the MC bulk was dispersed in three-fourth volume of demineralized water above 90 °C. Then, one-fourth of the volume of cold demineralized water was added and stirred for about 30 min. The final solution was stored at 4 °C, for 24 h, in order to ensure proper hydration of the polymer [24].

### 2.2. DSC

The measurements were carried out using a Pyris 1 DSC, Perkin Elmer (Waltham, MA, USA) differential scanning calorimeter in non-isothermal mode at constant heating and cooling rates of 2 K/min, in the temperature range –10–80 °C. In order to prevent water evaporation, dedicated stainless steel hermetic pans were used. As the heat flow of the thermal effects from MC was small and the hermetic pans have a low thermal conductivity, in order to increase the signal-to-noise ratio, the samples were of a higher weight, 70–75 mg, and they were measured with reference to a water sample of similar weight, which provided a similar heat capacity of sample and reference. At least three samples of each concentration were measured and, further, the most representative ones were measured during 10 heating–cooling cycles and the measurements were averaged. This further improved the signal. In order to reduce the artefacts from water reference, for the baseline, water versus water thermograms were recorded. 

Detailed analysis of the DSC heating scans was performed, in order to provide information on the kinetics of the transitions. First, the thermal effects were obtained from the heating scans by subtraction of the polynomial of the fifth order. Further, deconvolution of the thermal effects was performed using the asymmetric double sigmoidal function. Thus, the obtained separated thermal effects were analyzed, providing information on the heat exchanged, *ΔH*, the temperature of the beginning of the effect, *T_onset_*, and the transition rate, *k*. *ΔH* was obtained by integration of the thermal effect and the *T_onset_*was determined at the cross-section of the tangent at the half-height of the DSC peak, with the horizontal baseline. The transition rate, *k*, was determined as the reciprocal of the time of the half transition with respect to its start time.

### 2.3. DMA

Several concentrations of aqueous MC solutions (1.75–10 wt.%) were analyzed using DMA, Anton Paar Physica MCR301 rheometer (Anton Paar Physica, Graz, Germany). The kinetics of MC crosslinking was investigated by measuring the viscoelastic properties (storage, *G*′, and loss modulus, *G*″) of solutions at 37 °C in the oscillatory shear regime in a limited time range. Before the isothermal step, the samples were heated from 20 °C at a heating rate of 2 °C/min. The analysis was performed using cone-plate geometry (diameter—39.9 mm, angle 0.989°, and truncation 47 µm) and small-amplitude sinusoidal deformation (0.1% strain and 1 Hz frequency). A cone-plate geometry equipped with a dedicated solvent trap that prevented water evaporation from the solution was used. In some publications a silicone oil was used to minimize this effect (e.g., [25]), however, in this study, the silicone oil interfered with the MC crosslinking process.

All concentrations were analyzed 3–4 times. The measured *G*′ values were approximated with the biphasic dose-response function, belonging to the sinusoidal type of function, which was also used in the studies of progress of crosslinking by Maryanski and Dufresne [26,27]. Usually, this kind of function is used to show how an organism reacts to an applied drug dose, simulative agent, or stress. In our case, the use of this function allowed us to present how the various concentrations of MC influence their thermal crosslinking at a constant elevated temperature. Afterwards, the fitting curves were always extrapolated to the saturation plateau at a longer time (166 min) and then averaged. The kinetics of crosslinking was estimated from the time derivative of the averaged *G*′, which allowed for the definition of the maximum rate of increase of *G*′, taken as the maximum of the crosslinking rate of hydrogel. In order to estimate the hydrogel stiffness, *dG′/dt* were integrated, leading to the determination of the final storage modulus of hydrogel.

In order to compare the crosslinking kinetics from both methods (DMA and DSC), we determined the crosslinking rate, *k*, from the time of half transition, similar to the DSC case. We used the start of the crosslinking as the zero time-point, which differed from the procedure applied in the case of the time derivative of the *G*′ method, for which the time was measured when the temperature reached about 37 °C. The start of the crosslinking, *t_onset_*, was determined as the intersection of the tangent to the baseline before the start of the thermal effect and the tangent to the rising part of the thermal effect.

### 2.4. Biological Tests

#### 2.4.1. Cellular Cultures

Biological tests were carried out using mouse fibroblasts L929 line (Sigma-Aldrich; Merck Millipore, Darmstadt, Germany). The cells were cultured in 75 cm^2^ flasks in 89% High Glucose Dulbecco’s Modified Eagle’s Medium (DMEM), 10% fetal bovine serum (FBS), and 1% antibiotics, and were incubated at 37 °C in a 5% CO_2_ environment. The culture medium was changed every three days. A culture flask with 70% of cell confluency was used in experiment. L929 cells were seeded as follows—the cells were harvested by washing with phosphate buffer saline (PBS), then 5 mL of 0.05% (0.25%, diluted five times with PBS) trypsin solution was added to the flask and placed for 3 min in the incubator. Following this step, the side of the flask was gently tapped in order to ensure the detachment of the cells from the bottom of the flask. A 10 mL of culture medium was added to the harvested cells and then centrifuged for 5 min at 100× *g* (times gravity, the unit of relative centrifugal force (RCF)) at room temperature. The collected pellet was resuspended within the culture medium, followed with dilution to the required cell density. L929 cells were seeded in a 48-well plate at a density of 5000 per well in 500 μL of growth medium and were left to attach to the plate in the incubator at 37 °C, in a 5% CO_2_ environment.

#### 2.4.2. MC Preparation

For these tests, the MC powder was added to the proper concentrations and was sterilized using UV light for 30 min (every 10 min, the powder was shaken to provide a homogenous sterilization). The MC aqueous solutions were prepared in water at concentrations of 1, 2.5, and 5 wt.%, respectively. The samples were added to the cells in a volume of 25, 50, and 100 μL. The obtained solutions of MC and the cells were incubated at 37 °C, overnight, to induce MC crosslinking. After crosslinking, 500 μL of the crosslinked MC with the appropriate cell amounts were added and incubated at 37 °C. The cells seeded on tissue culture plastic (TCP) served as a control.

#### 2.4.3. Cytotoxicity Tests

Cytotoxicity of the MC aqueous solutions was evaluated in direct contact with the cells via the metabolic activity assay, using the PrestoBlue agent. In the PrestoBlue test, blue resazurin reagents are reduced to resorufin. As a result of these red fluorescent compound cells, viability might be measured quantitatively and visually (absorbance, by observing fluorescent outputs of reduced resorufin).

After three days of incubation, the culture medium was removed and the PrestoBlue–DMEM solution (1:10 *v*/*v*) was added. The plate was incubated for the next 90 min. As a final step, 100 μL of the solution from each well was transferred to a 96-well plate. Fluorescence was read with excitation/emission 530/620 nm filters, using the Fluoroscan Acent FL instrument (Thermo Fisher Scientific, Waltham, MA, USA). The obtained signals were compared with the fluorescence of PrestoBlue in wells without cells, which served as blank samples without any metabolic activity, and as wells of control (with 100% metabolic activity).

#### 2.4.4. Cell Morphology

In order to evaluate how MC solutions affect the cells morphology in direct contact, scanning electron microscopy (SEM) and fluorescent images were taken. In both experiments, the samples were placed in a separate 48-well plate. L929 cells were seeded in cell density 3000 per well and were subjected to 5 days of long culture.

SEM images were obtained on the dried samples. Preparation for SEM assumed the following steps—the plate was taken out of the incubator, samples with cells were washed using PBS and treated with glutaraldehyde (GA) for 2 h. After that, they were washed with PBS three times, in order to remove the residues of GA. Then, the samples were dehydrated with a series of ethanol solutions (using increasing EtOH concentrations from 50 to 100 wt.%). After that, they were washed with EtOH:HDMS (hexamethydisilazane) solutions (1:2, 2:1 (*v*/*v*)) and 100% HDMS in which they were stored to dry. In each solution, the samples were left for 20 min. Finally, the dried samples were sputter-coated with 7 nm of gold. SEM imaging was carried out with a JEOL JSM-6390LV.

Fluorescent microscope (FM) imaging required fixing of the samples and staining of the cytoskeleton. Just before fixing and after the next steps, every sample was washed with PBS. In order to fix the samples, they were immersed in 3% formaldehyde for 20 min and then in 0.1% Triton X 100 for 5 min, to obtain permeable cell membranes. Then, they were put in a staining solution, ActinGreen (ThermoFisher Scientific, Waltham, MA, USA), for 30 min. The wet samples were placed on the standard glass plates and covered with a thin microscopic glass, in order to prevent drying of the sample. All images were taken using a Leica AM TIRF MC.

## 3. Results and Discussion

### 3.1. DSC

**Figure 1:**
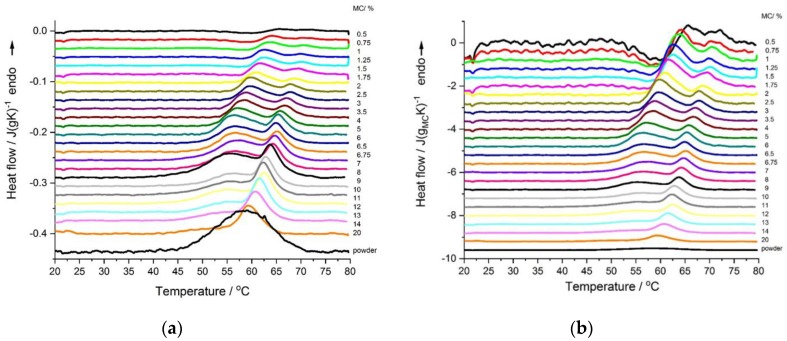
DSC heating scans of MC solutions with various concentrations (indicated)—(**a**) heat flow normalized to the sample weight, and (**b**) heat flow normalized to the methylcellulose (MC) weight. Curves subtracted using polynomial. For comparison, the curves are shifted in *y*-axis.

The DSC heating thermograms of MC solution showed quite complex thermal behavior and complex changes of the effects with varying MC concentration. For better understanding of the effect of the MC content on the thermal effects, all thermograms are presented in Figure 1a,b, as normalized to sample weight and normalized to MC weight, respectively. Except for the MC powder, all thermograms generally presented two endothermic effects. Both effects were more evident for low MC concentrations (0.5–8 wt.%). The low-temperature effect dominated over the high-temperature one, as far as the MC concentration decreased. It was seen that both endothermic peaks shifted to lower temperatures with increasing MC concentrations, (Figure 1) with the low-temperature peak becoming broader, which was particularly evident at MC concentrations of above 8 wt.%. (Figure 1a). MC in the powder form (~100 wt.%) only showed one broad endothermic peak (Figure 1a).

In Figure 1b, it could also be seen that, for the lowest MC concentrations, i.e., <wt.%, there appeared an additional effect, which was exothermic and preceded the low-temperature endothermic effect. The exothermic effect became stronger with a decrease in the MC concentration. 

Based on the literature [16], we interpreted that the low-temperature endothermic effect was due to the destruction of the water cages and the high-temperature endothermic effect was due to the MC thermal crosslinking. For the exothermic effect observed in concentrations <2 wt.%, we interpreted it as due to the formation of water cages around methoxy groups. A more detailed explanation is given below.

**Figure 2:**
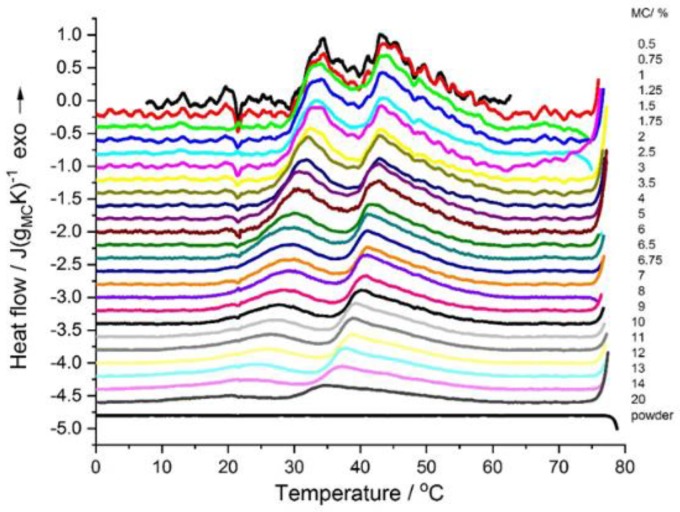
DSC cooling curves of the MC solutions with various concentrations (indicated) normalized to the MC weight. Curves subtracted using polynomial. For comparison, the curves are shifted in the *y*-axis.

The DSC cooling thermograms showed two exothermic effects (Figure 2), a high-temperature effect dominating over the low-temperature one. These effects gave evidence of the reverse processes, i.e., first decomposition of the three dimensional hydrogel network followed by water “incorporation” into the polymer structure. Both effects appeared in the temperature range of 30–55 °C. The most significant finding here was that the MC physical crosslinking was fully thermoreversible. Additionally, for MC concentrations <2 wt.%, the reverse effect of the destruction of water cages, which should be endothermic, was not observed, however, the occurrence of this process was evident from the repeating appearance of the exothermic peak upon a subsequent heating scan. Detailed analysis of the cooling scans would provide more insight into this question, however, the current study was devoted to the crosslinking kinetics and the cooling scans were not studied in detail. Additionally, it might be noted that, for the MC powder, the crosslinking was non-reversible as, upon cooling and subsequent heating scans, no thermal effect was observed (Figure 2).

**Figure 3:**
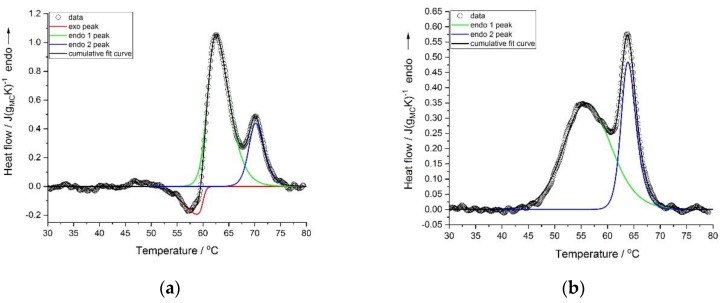
Deconvolution of the thermal effects registered during heating for MC concentrations—(**a**) 1.5 wt.% and (**b**) 9 wt.%.

Deconvolution of the thermal effects was performed using the asymmetric double sigmoidal function. The examples of the deconvolution of the heating scans with and without the exothermic effects are presented in Figure 3a,b, respectively.

**Figure 4:**
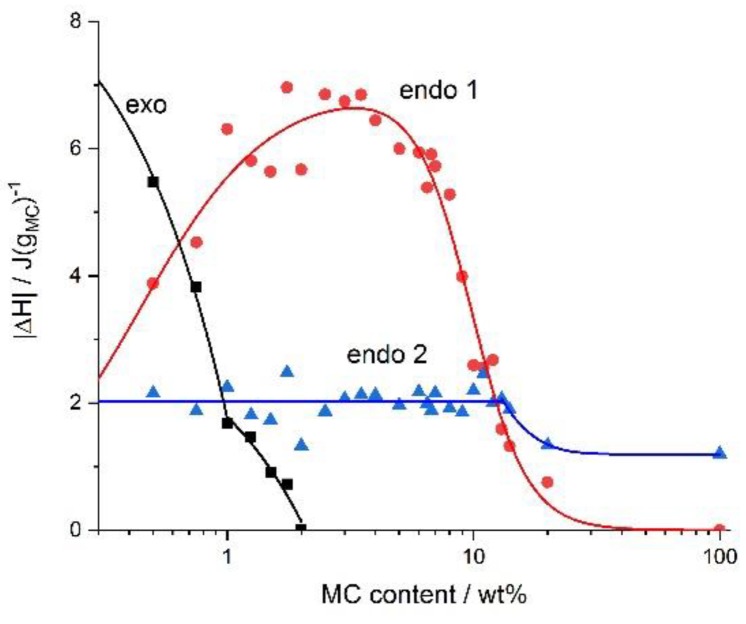
The absolute value of the heat exchanged upon heating during the exothermic, the low-temperature (endo 1) and the high-temperature (endo 2) endothermic effects versus the MC content.

**Figure 5:**
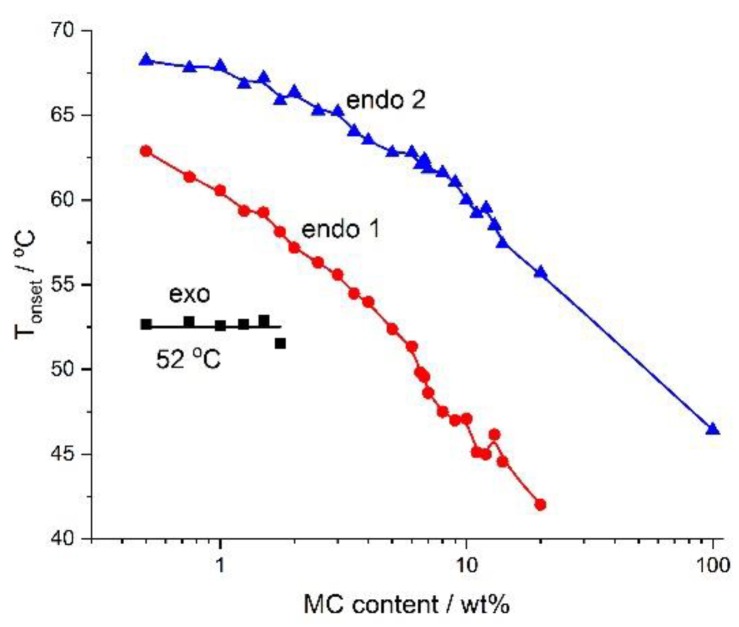
Phase diagram for various concentrations of MC constructed using the onset temperature.

**Figure 6:**
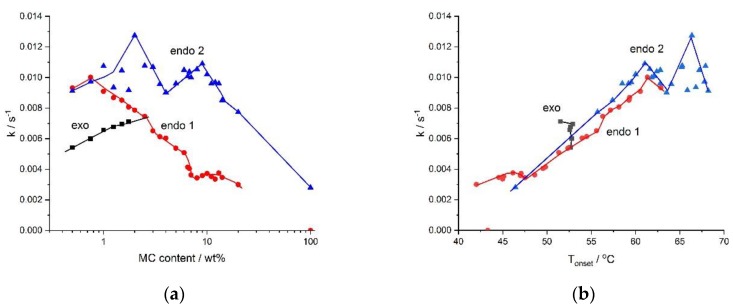
The rates, *k*, of water cages formation (exo), their destruction (endo 1), and of crosslinking (endo 2), as a function of—(**a**) the MC content and (**b**) the temperature, *T_onset_*.

Detailed analysis of the deconvoluted peaks provided quantitative information on the effect of the MC concentration on the kinetics of the processes. The results are presented in Figure 4, Figure 5 and Figure 6, showing the heat exchanged, *ΔH*, the temperatures at the start of the processes, *T_onset_*, and their rates, *k*, respectively. In Figure 4, it can be seen that the maximum of the low-temperature endothermic effect (endo 1) was ca. 7 J/g_MC_, whereas for the high-temperature endothermic effect (endo 2), the maximum heat was ca. 2 J/g_MC_. In case the water cages were destructed (low-temperature endothermic effect), the highest heat levels were seen for the medium range of MC concentrations, i.e., 2–6 wt.%. In the case of the crosslinking process (high-temperature endothermic effect), the heat was constant up to 14 wt.% of the MC. At the highest MC concentrations, both heat levels dramatically decreased the low-temperature endothermic effect to 0 and the high-temperature endothermic effect to ca. 1 J/g_MC_. In the left panel of Figure 4, in the low MC concentration range of <2 wt.%, the heat of the low-temperature endothermic effect decreased with decrease in the MC content. It was accompanied by the appearance of the exothermic effect, whose heat levels increased with a decrease in the MC content. Interestingly, the change in the exothermic heat showed two ranges of MC content dependence with a boundary at 1 wt.%. 

There are some ideas to explain the genesis of the endothermic nature of the MC crosslinking during heating. Sekiguchi et al. [28] suggested that crosslinking might not only involve hydrophobic interactions. Other interactions might also be responsible for the double MC crosslinking process. Our explanation of the observed phenomena based on the DSC results during the heating of the MC solutions was the following. In the ambient conditions, MC was characterized by prevalence and dominance of hydrophilic interactions in the solution, such as water–water interaction and these same interactions between the hydrophilic parts of MC and the water molecules. Additionally, in the solution, the water molecules might have created ordered structures, i.e., water cages around the hydrophobic regions of MC, and intramolecular interactions between the polymeric chains were also formed. Formation of water cages around the methoxy groups was accompanied by heat release and entropy reduction, which was manifested by the exothermic effect preceding the endothermic effects for highly diluted MC solutions. The exothermic effect disappeared at higher MC concentrations. It is possible that in more densely packed polymeric networks, hydrophobic aggregates were previously created at lower temperatures than what was caused by the limited available space in the solution and the dominance of the polymeric phase over the solvent.

While heating, an appropriate amount of energy (heat) is delivered to the system, the water cages are destroyed, and the methoxy groups are released, and the number of ordered form of water molecules start to change. Heat was consumed to make the layer of the water cages thinner, increasing disordering and, thus, entropy (ΔS > 0). This process was accompanied by the low-temperature endothermic effect.

Soon after the destruction of the water cages, the exposed methoxy groups in the MC chains underwent cross-linking due to intra- and intermolecular hydrophobic interactions. As we mentioned previously this step was considered to be the high-temperature endothermic effect.

The maximum effect of the heat on the water-cage destruction (low-temperature endothermic effect), at a particular range of MC concentration, in all probability could have been related to the presence of two limitations. In a dilute MC solution, the MC molecules might form micelles, providing a limited number of the methoxy groups to the solution and to the water cages around these molecules. On the other hand, at relatively high MC concentrations, the chains become densely packed, and their molecular mobility is limited, which also affects the mobility of the water molecules, and results in a reduction of the low-temperature endothermic effect. In the case of a high-temperature endothermic effect, the constant value of the heat at low and medium MC concentrations, might suggest similar employment of the methoxy groups, which at dilute MC solutions would create separated molecules that form micelles and at high-enough MC concentrations would form a hydrophobic network. Decrease in the heat of the high-temperature endothermic effect, at above 14 wt.% of MC was understood to be due to the aforementioned limited molecular mobility.

The dependence of the exothermic heat on the MC concentration in Figure 4, showed an abrupt change at 1 wt.% of the MC content. This behavior indicated the existence of some boundary, above which the exothermic heat weakly depended on the MC concentration and below which it depended strongly on the MC concentration. This might suggest the boundary between semi-dilute and dilute solution, respectively, however, this assumption needs further investigations. 

Figure 5 presents the dependencies of *T_onset_* and MC concentrations, which might serve as a phase diagram. It is rather clear that below the *T_onset_* of the low-temperature endothermic effect, there existed a one-phase solution in the form of sol. Between the low-temperature and the high-temperature endothermic effect there was a sol–gel transition, while above the high-temperature endothermic effect there might have existed a cross-linked polymer (gel). According to [29], the MC gel remains stable up to 80 °C, where phase separation takes place. Additionally, Figure 4 shows the dependence of the *T_onset_* of the exothermic effect, which showed a constant value. It suggests the existence of two sub-areas in dilute or semi-dilute sol, characterized by a lack of and formation of water cages below and above 52 °C, respectively.

The kinetics of the processes, described using rate, *k*, determined as the reciprocal of the half time of the process is presented in Figure 6. Figure 6 illustrates the effect of the MC concentration on the rates of the processes. From Figure 6a, it is evident that the low- and the high-temperature endothermic processes, as well as the exothermic one, differed in rates. The rate of destruction of the water cages (low-temperature endothermic effect) was seen to decrease with the MC concentration. This might have been due to a decrease in the MC molecular mobility. The rate of the formation of the water cages (exo) was seen to increase with MC concentration, confirming the reinforcing role of MC molecules in the creation of water cages. The rate of the crosslinking process (high-temperature endothermic effect) showed a reduction in the high MC concentration range. This was obviously due to a decrease in the molecular mobility. It was accompanied by a decrease in *ΔH* in Figure 4, indicating the formation of an imperfect crosslinked network of low density. At low and medium MC content, the crosslinking rate showed generally comparable values, however, it might be noted that, there existed two maxima at 2 wt.% and 9 wt.% of MC, separated by a local minimum at 4 wt.% of MC. As shown in Figure 4, up to 14 wt.% of MC, there was a constant value of *ΔH*, indicating the formation of highly dense crosslinking network. Thus, these kinetic features indicated the existence of two regimes in the MC crosslinking kinetics, which were expected to be controlled by different mechanisms. Due to these features, there were predicted differences in the structure of the crosslinked networks, however, further studies are necessary. 

The kinetics was additionally affected by the temperature of the process. It can be seen in Figure 6b, that the processes occurring at higher temperatures were faster than those at lower temperatures. We anticipated that there was a similar effect of various molecular mobility, being dependent in this case on temperature. Interestingly, all rates showed similar values at the same temperature. Moreover, it might be noticed that the rates of crosslinking and destruction of the water cages showed a similar abrupt drop at 61 °C and in case of the former rate, it also showed two additional abrupt changes at 63 °C and 66 °C. This coincidence suggested that the two crosslinking mechanisms proposed above might have been temperature activated. 

### 3.2. DMA

Analysis of the G′ and G″ as a function of time was used to determine the crosslinking kinetics of thermoresponsive materials. In most cases described in the literature, e.g., [29], the G′ and G″ curves intersected each other at a particular time with further domination of G′ over G″. While the G′ curve was below the G″ curve, the material presented viscous behavior characteristic for liquids. The intersection was the characteristic point, which was usually taken as the time (or temperature) of crosslinking [5]. The dominance of G′ over G″ indicated the elastic behavior of the material [29].

There is a specific group of materials in which the intersection of G′ and G′’ has not been detected. We observed such a situation for the isothermal measurements of the MC aqueous solutions. During every measurement, the G′ was higher than G″, showing the weak elastic character of MC aqueous solutions, even at ambient conditions. Consequently, in order to estimate the crosslinking kinetics, only G′ was analyzed as a function of time.

**Figure 7:**
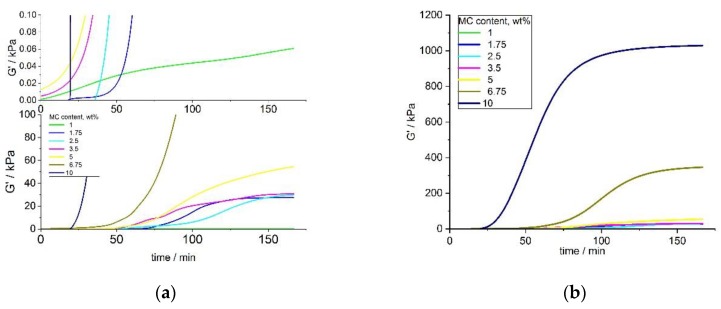
G′ versus time at 37 °C for several MC concentrations—(**a**) enlarged view of the low G′ range, and (**b**) the whole G′ range.

Results for small concentrations of MC ranging between 1 and 5 wt.% and the full range of MC concentrations 1–10 wt.% (shown in Figure 7a,b) indicated a sigmoidal growth of G′, which was non-ideal, particularly for lower concentrations. Different onset time of crosslinking among the various concentrations of MC was observed. The G′ of MC at the lowest concentration of 1 wt.% showed very different behavior, compared to the solutions with higher concentrations, with a very slow growth and the lowest final value. The final values of G′, for small MC concentrations (1–5 wt.%), increased with the MC concentration. An analysis of G′ at the full range of MC concentrations (Figure 6b) showed a smoother G′ increase for higher MC concentrations (6.75 and 10 wt.%), with a clearly marked stabilization at the end of the crosslinking. As expected, an increase in the G′ values with an increasing concentration for 5 and 10 wt.% of MC was also observed.

After heating up to 37 °C, for a long time, the MC aqueous solution showed a delayed thermal destruction of the water cages, resulting in an unstable increase of G′. The analysis of the G′ and *dG′/dt* curves clearly indicated that the crosslinking rate and the final G′ value depended on the temperature and polymer concentration. However, the heating time also played a key role here. As mentioned before, diluted MC aqueous solutions need prolonged heating to destroy water cages and initiate a polymeric chain rearrangement. Since the number of methoxy groups is partial and the distance between them is prominent, the greater amount of time that is needed to ensure a contact between them results in a hydrophobic bond formation. Solutions with the highest MC concentrations showed an instant G′ increase corresponding to the first step of crosslinking. In a denser polymeric network, there are partially formed hydrophobic interactions, and hence it results in less water cages breakage. As a result, G′ increase was more stable and needed less time to form fully crosslinked network. Another reason for the multiple effect of crosslinking for small concentration might be the temperature, which was not high enough to induce crosslinking. Since the hydrophilic solvent dominated in the solution, it was difficult to destroy all of the water cages. Some of the methoxy groups were entrapped and could not form a fully formed hydrophobic network. Probably for such concentrations, a higher temperature was needed to induce crosslinking and increase the rate of the process. For the higher MC concentrations, the polymeric chains were closer to each other and it was feasible to form hydrophobic interactions. Denser packed MC chains tended to make bridges with water molecules [30]. In a partially cross-linked network, aggregation occurred at sufficiently high temperatures, when it was easier to break down water cages at higher concentrations.

**Figure 8:**
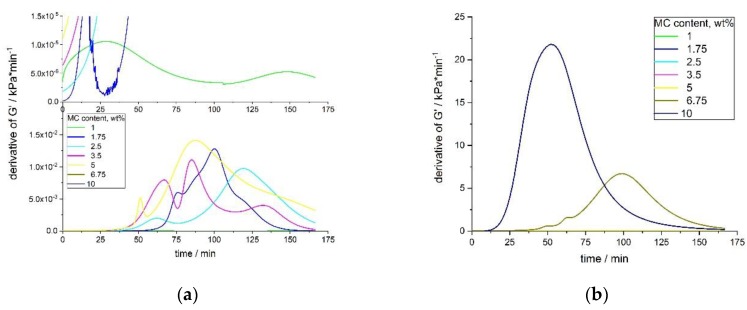
dG′/dt versus time for several MC solutions—(**a**) enlarged view of low MC concentrations, and (**b**) the whole dG′/dt range.

The time derivatives of G′ for low MC concentrations showed a complex multimodal character, which was a consequence of the non-ideal sigmoidal character of the G′ growth (Figure 8a,b). For every sample, the height of the first maximum was smaller than the second one, and appeared later than 50 min of the measurement. The height of the second maximum was always the highest and appeared in the time range of 80–120 min of the measurement. In some samples (1.75 and 3.5 wt.%), there was also a third smallest maximum, appearing at the latest stage of the process.

With an increasing MC concentration, the process became more homogenous and there might have been only one maximum in the time-derivative of the G′ curve. Moreover, the process became faster (according to the height of the peak and its time position) (Figure 8b). Although, MC at 6.75 wt.% still showed two maxima in the *dG′/dt*, the first maximum was practically negligible, as compared to the second large one. MC at 10 wt.% showed only one maximum at 52 min. The onset time of the crosslinking differed for various concentrations, being shorter at higher concentrations. This was especially evident for the 6.75 and 10 wt.% of MC.

**Figure 9:**
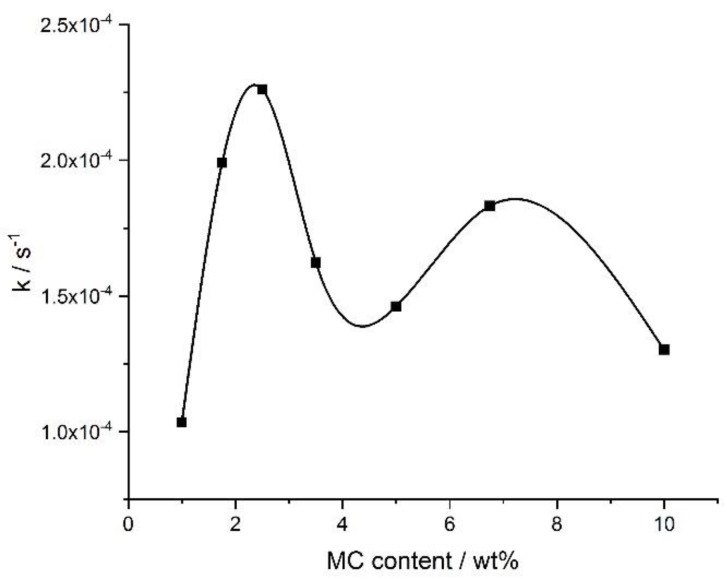
Crosslinking rate, *k*, determined by DMA, versus the MC content.

Figure 9 shows the crosslinking rate, as determined from the DMA results, as a function of the MC concentration. There were two maxima at ca. 3 wt.% and ca. 8 wt.% with the local minimum in between, which was qualitatively similar as obtained by the DSC for the crosslinking process, manifesting as the high-temperature endothermic effect (compared with the ‘endo 2’ in Figure 6a).

Table 1 shows the final maximum values of G′, which were determined as integrated extrapolated G′ derivatives. These values were analyzed and compared to the G′ of various human tissues (Table 2). G′ for the lowest MC concentration (1 wt.%) was significantly lower, as compared to others. MC of 1.75–3.5 wt.% showed similar values, and above these concentrations the G′ increased significantly. 

The final G′ versus the MC concentration dependency was just as predicted. The higher concentration resulted in higher final hydrogel stiffness. The obtained results suggest that, with regards to the viscoelastic properties, the MC aqueous solution might be a suitable material used as a scaffold for tissue engineering. In order to definite a dedicated application of the studied MC concentrations, the obtained results were summarized and compared to the G′ of various human tissues (Table 1 and Table 2). According to the tables, the stiffness of MC in concentrations of 1.75–3.5 wt.% might be used for spinal cord therapies as a scaffold or drug/cell delivery system. MC at the concentration of 6.75 wt.% presents similar mechanical properties (G′ value) to the human meniscus. MC at a concentrations of 10 wt.% shows high values, which according to Table 2, might be suitable for cartilage applications.

### 3.3. Biological Tests

**Figure 10:**
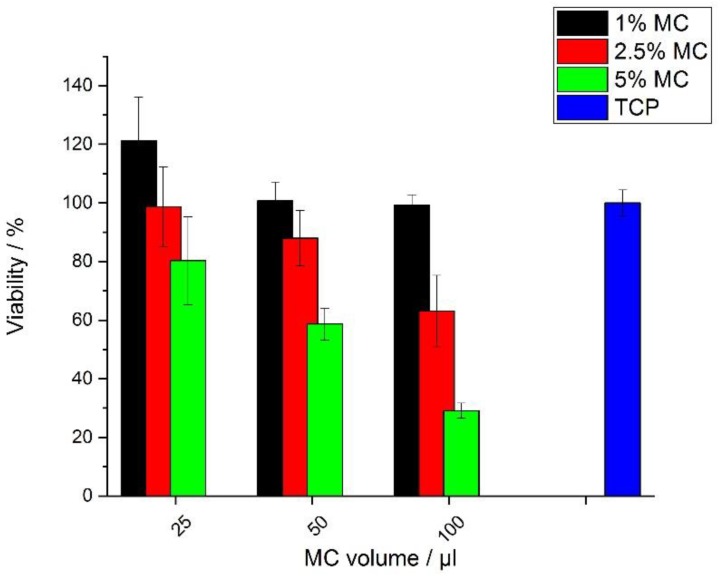
PrestoBlue cell proliferation results for the MC hydrogel for several MC concentrations (after 3 days).

The cytotoxicity of the MC aqueous solutions was performed by the PrestoBlue assay after 3 days. Cells were directly exposed to 1, 2.5, and 5 wt.% of the MC aqueous solutions in volumes of 25, 50, and 100 µL. Biological tests including cytotoxicity and viability of the cells on MC hydrogels were carried out to assess its relevance in tissue engineering. For MC concentrations of 1 and 2.5 wt.%, the metabolic activity was about 90% of that of the untreated cells (Figure 10). For the lowest MC concentrations, each volume of added solution performed cell viability over 70%. MC of 5 wt.% achieved this cell viability in only one volume of 25 µL. Both of these values were ≥70%, which according to UNI ISO 10993-5 standard is considered to be non-toxic for cells. At higher MC concentrations, the metabolic viability was rather low (Figure 10).

**Figure 11:**
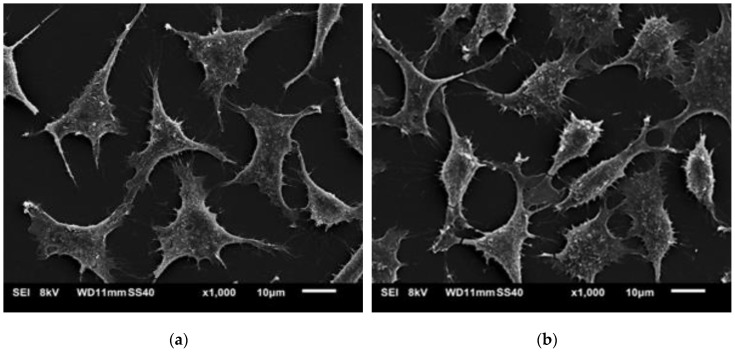
SEM of L929 cells on (**a**) tissue culture plastic (TCP) (control) and (**b**) hydrogel containing 1 wt.% of MC (50 µL volume).

**Figure 12:**
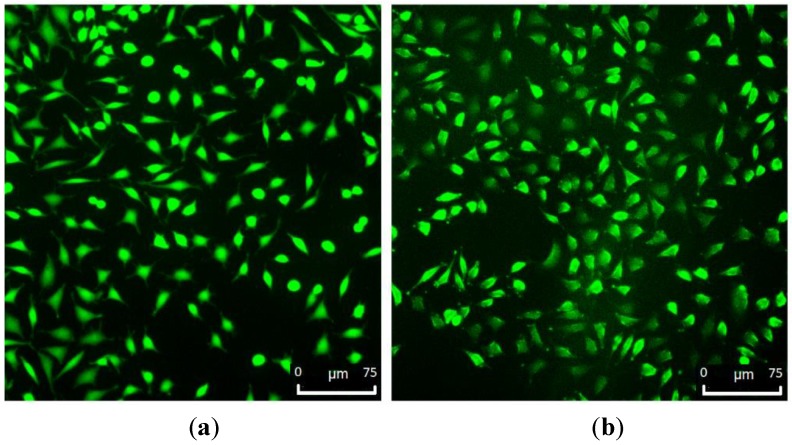
Fluorescence microscopy of stained L929 cells on—(**a**) TCP (control) and (**b**) hydrogel containing 1 wt.% MC (50 µL volume).

SEM and fluorescence images (Figure 11 and Figure 12) showed that the L929 fibroblasts penetrated the MC hydrogels to some extent. It was especially visible in fluorescent imaging that the density and viability of the cells were higher than that of the control. The varying FM cell image focus indicated different cell penetration depths into the hydrogel (Figure 12b). This effect was less visible on the SEM images (Figure 11b).

Our results indicated that the L929 cells seeded on top of the gels remained viable with no indication of cell death throughout the culture duration. However, the different concentrations and added volumes of MC influenced the viability, metabolic activity, and morphology of this cell line. It was evident that MC with low MC concentrations (below 2.5 wt.%) did not cause cytotoxicity. At these concentrations an appropriate pH and availability of oxygen were ensured. However, at MC concentrations ≥5 wt.% the material seemed to be toxic for fibroblast. It was probably the effect of the acidic pH and a limited availability of oxygen. The limited availability of oxygen could have been a result of the high viscosity of the hydrogel. At these MC concentrations, the cells collapsed and did no longer had the possibility to conduct any metabolic activity. It was also observed that the amount of added hydrogel significantly influenced the cells viability. The more added hydrogel, the higher toxicity, especially, at higher MC concentrations. It was probably caused by the non-controlled pH in the MC aqueous solutions. However, in the future, we plan to study a mixture of MC with PBS, in order to control the pH of the solution, which is expected to increase cell viability on the investigated materials.

## 4. Conclusions

We systematically studied a broad range of MC concentrations in aqueous solutions, to explore in details, the crosslinking kinetics, thermal, viscoelastic, and biological properties. We have assessed the real value of MC as a material for tissue engineering, including its advantages and disadvantages. For this evaluation DSC, DMA measurements, and biological analyses were carried out. It was evidenced by DSC that the crosslinking of MC was a reversible two-stage process, manifested by the appearance of two endothermic effects related to the destruction of water cages around methoxy groups, followed by crosslinking via formation of hydrophobic interactions between methoxy groups in the polymeric chains. Moreover, at relatively small MC concentrations (below 2 wt.%), there appeared to be an additional exothermic process preceding the endothermic ones, which was most probably related to the formation of the water cages. Our DSC studies allowed us to construct the phase diagram for the studied MC aqueous solutions. The crosslinking kinetics of the MC solution, as determined by the DSC and DMA, was found to be quite complex, indicating three regimes. At MC higher than 13 wt.%, the crosslinking rate dramatically decreased with the MC concentrations, which was due to a reduced molecular mobility. At MC concentrations below 13 wt.%, there seemed to be two regimes evidenced by two maxima at 2 wt.% and 9 wt.%. In these regimes, the crosslinking mechanisms might differ and, moreover, the change of the mechanism might be temperature activated. These mechanisms need to be further investigated. Nevertheless, the obtained results allowed the selection of MC concentrations that might meet the tissue engineering expectations. Since MC at concentrations in the range of 1.7–3.5 wt.% and at ca. 9 wt.% showed the fastest crosslinking, these concentrations could be attractive for tissue engineering applications. Tissue engineering is rather demanding, it requires looking not only from the crosslinking rate perspective, but also from a mechanical and biological point of view. For lower MC concentrations, the final storage modulus (DMA) s comparable to native human tissues such as liver [13], spinal cord [14], or cartilage [33]. From biological tests, it was found that at low concentrations, MC provided a good cellular response and non-toxic environment. It was highly probable that it would be necessary in the future to modify MC solutions in order to improve their properties. Nevertheless, we are certain that, due to the minimally invasive manner through which these materials were introduced into the body, and because of the mechanical and the biological properties of MC, relatively small concentrations of this material can have a great potential as injectable scaffolds for tissue engineering.

## Figures and Tables

**Table 1:** Final storage modulus, G′, determined for several MC concentrations.

MC Concentration [wt.%]	Final G′ [kPa]	SD
1	0.06	0.004
1.75	27.4	0.53
2.5	29.9	1.56
3.5	30.7	1.44
5	54.4	7.57
6.75	346.2	10.92
10	1029.3	316.03

**Table 2:** G′ of various human tissues.

Tissue	G′ [kPa]	Ref.
Brain:		[31]
Cerebellum	1.77–2.03	
Cerebrum	2.34–2.64	
White matter	2.41–12.1	
Grey matter	2.34–6.6	
Skin (Human skin fibroblasts)	0.14–0.26	[32]
Meniscus:	280–330	[12]
Lateral Posterior	770	
Lateral Mid body	480	
Liver	37–340	[13]
Spinal cord *	5–42	[14]
Articular cartilage (hip and knee)	1000–1700	[33]

* Shear modulus of the human spinal cord.
